# The Hierarchy of Defense Mechanisms: Assessing Defensive Functioning With the Defense Mechanisms Rating Scales Q-Sort

**DOI:** 10.3389/fpsyg.2021.718440

**Published:** 2021-10-15

**Authors:** Mariagrazia Di Giuseppe, J. Christopher Perry

**Affiliations:** ^1^Department of Surgical, Medical and Molecular Pathology, Critical and Care Medicine, University of Pisa, Pisa, Italy; ^2^McGill University Department of Psychiatry at the Institute of Community and Family Psychiatry, Jewish General Hospital, Montreal, QC, Canada

**Keywords:** defense mechanism, DMRS, Q-sort, assessment, personality, emotion regulation, psychotherapy, process-outcome

## Abstract

The psychodynamic concept of defense mechanisms is nowadays considered by professionals with various theoretical orientations of great importance in the understanding of human development and psychological functioning. More than half century of empirical research has demonstrated the impact of defensive functioning in psychological well-being, personality organization and treatment process-outcome. Despite the availability of a large number of measures for their evaluation, only a few instruments assess the whole hierarchy of defenses, based on the Defense Mechanisms Rating Scales (DMRS), which arguably offers an observer-rated gold standard of assessment. The present article illustrates the theoretical and methodological background of the DMRS-Q, the Q-sort version of the DMRS for clinical use. Starting from the definition and function of the 30 defense mechanisms included in the hierarchy, we extracted 150 items that captured a full range of defensive manifestations according to the DMRS theory. The DMRS-Q set is described in this paper with reference to the DMRS manual. Directions are also provided for using the DMRS-Q online software for the free and unlimited coding of defense mechanisms. After each coding, the DMRS-Q software provides a report including qualitative and quantitative scores reflecting the individual’s defensive functioning. Qualitative scores are displayed as the *Defensive Profile Narratives* (DPN), while quantitative scores are reported as Overall Defensive Functioning (ODF), defensive categories, defense levels, and individual defense mechanisms. Syntax for the scoring is displayed in the results and a clinical vignette of a psychotherapy session coded with the DMRS-Q is provided. The DMRS-Q is an easy-to-use, free, computerized measure that can help clinicians in monitoring changes in defense mechanisms, addressing therapeutic intervention, fostering symptoms decreasing and therapeutic alliance. Moreover, the DMRS-Q might be a valid tool for teaching the hierarchy of defense mechanisms and increase the observer-rated assessment of this construct in several research fields.

## Introduction

The psychodynamic concept of defense mechanisms, defined as automatic psychological mechanisms that mediate the individual’s reaction to emotional conflicts and to internal or external stressors (American Psychiatric Association, 2013; Perry, 2014), has been extensively studied since its first appearance in Freud’s psychoanalytic theory (Freud, 1894). After a century of clinical and theoretical work, and a quarter century of empirical research, an assessment of defense mechanisms was included in an Axis for the assessment of defense mechanisms in the DSM-IV (Cramer, 1987, 2015; Kernberg, 1988; American Psychiatric Association, 1994; Hoffman et al., 2016). The main contribution to the gold-standard approach to the study of defense mechanisms has been provided by the theory of defensive adaptiveness and the hierarchical organization of defense mechanisms proposed by Vaillant (1971, 1992) and operationalized by Perry (1990). In his extensive and valuable work, Vaillant described excellent clinical vignettes of defenses as they operate in real life – both in momentary examples, and those that recur over time – and integrated findings from several longitudinal studies demonstrating the evolution of defense mechanisms over the life cycle. With the development of the *Defense Mechanisms Rating Scales* (DMRS), Perry has provided a comprehensive, accurate and valid observer-rated methodology for assessing individual’s defensive functioning based on the whole hierarchy of defense mechanisms (Perry and Henry, 2004). In recent years, the authors of this paper have adapted the DMRS theory to additional assessment methods, by developing both the Q-sort version (DMRS-Q; Di Giuseppe et al., 2014) and the self-report version (DMRS-SR-30; Di Giuseppe et al., 2020a) of the DMRS. Our main aim was to provide new measures based on the DMRS theory of defense mechanisms applicable in different clinical or research contexts, without the requirement of training for their valid and reliable use (Békés et al., 2021; Conversano and Di Giuseppe, 2021). In this article, we describe theoretical background, coding procedure, scoring system and results interpretation of the DMRS-Q, a computerized observer-rated Q-sort for the assessment of defense mechanisms in clinical setting.

### The Hierarchy of Defense Mechanisms

**FIGURE 1:**
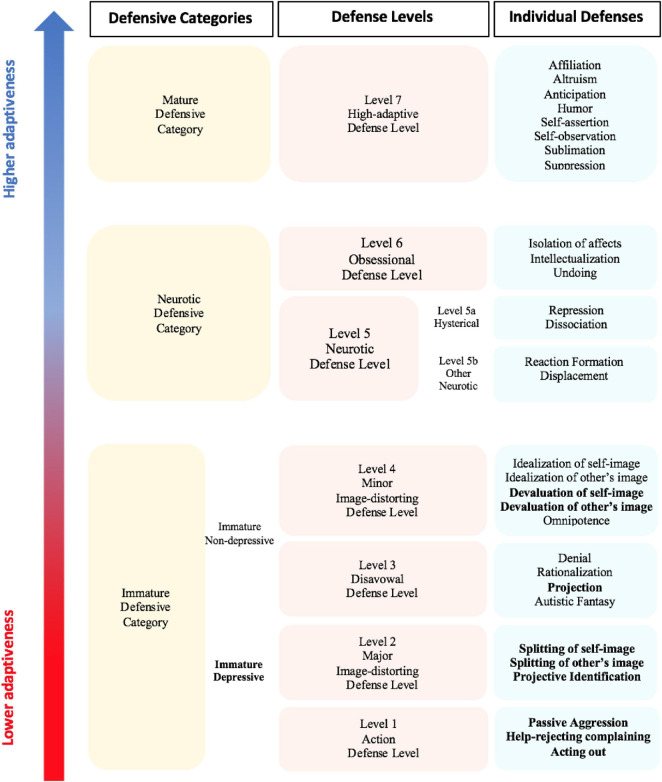
The DMRS hierarchical organization of defensive categories, defense levels and individual defenses. Table adapted from Perry and Bond (2012), Table 1. So-called depressive defenses are in bold.

All DMRS-based measures refer to the generally accepted hierarchy of defense mechanisms (American Psychiatric Association, 1994, 2013; Hoglend and Perry, 1998; Lingiardi et al., 1999; Drapeau et al., 2003; Hilsenroth et al., 2003; Perry, 2014; Di Giuseppe et al., 2019, 2021; Tanzilli et al., 2021). A graphical summary of the hierarchy of defense mechanisms is shown in Figure 1.

This hierarchy describes 30 defense mechanisms organized into seven defense levels, each of which has some general functions that the constituent defenses share in how they protect the individual from anxiety, or a sense of threat from internal or external sources, or conflicts.

In addition to the seven defense levels, there is level 0, describing defensive dysregulation, the so-called Psychotic Defenses Level. Defenses belonging to this defense level are not included in the DMRS manual although they can be assessed using another DMRS-derived measure, the Psychotic-DMRS (P-DMRS; Berney et al., 2014; Boldrini et al., 2020). Defense levels can be further organized into three defensive categories of relatively similar degree of maturity, often used for describing in summary the individual defensive functioning. The three defensive categories, from the least to the most adaptive, respectively, include immature, neurotic and mature defenses. The immature defensive category is the most populated and includes all defenses belonging to action, disavowal and both image distortion defense levels. This defensive category can be further divided into two subcategories. The first is named depressive defenses, including acting out, help-rejecting complaining, passive aggression, splitting of self-image, splitting of other’s image, projective identification, projection, devaluation of self-image, and devaluation of other’s image. The second subcategory is the non-depressive defenses, including denial, rationalization, autistic fantasy, omnipotence, idealization of self-image, and idealization of other’s image. Greater reliance on immature defenses informs on the subject’s defensive vulnerability and his or her scarce awareness of both emotional and cognitive sides of internal conflicts or external stressful situations. These defenses inhibit awareness of unacceptable ideas, feelings, and actions, bypassing them to protect oneself from feeling threatened.

The neurotic defensive category represents the middle-range of adaptiveness and includes all defenses belonging to neurotic and obsessional defense levels. High use of these middle-range defenses describes the individual’s ability to deal with either the emotional or the cognitive side of internal or external stressors, which can be handled one at a time. These defenses help the individual in keeping out of awareness parts of the conflict (e.g., associated feelings, desires and thoughts), which would generate intolerable anxiety if perceived as an integrated psychological experience. Finally, the mature defensive category corresponds to the high-adaptive defense level and includes the most adaptive defense mechanisms, which overlap with what are called positive coping strategies in other theoretical frameworks. High use of mature defenses fosters the integrated and partially aware experience of feelings, ideas, desires and thoughts associated to an internal conflict or external stressful situation. These defenses help the individual in dealing with his or her psychologically stressful experiences by integrating affects with ideas, therefore optimizing and possibly resolving the internal or external cause of distress (Vaillant, 1977, 1992). This tripartite model of DMRS hierarchical organization of defenses is often used for summarizing the defensive maturity of an individual by looking at the proportional scores obtained in each of the three defensive categories.

For a deeper understanding of individual’s defensive functioning, the seven defense levels can be used as the generally accepted hierarchical organization of defense mechanisms (American Psychiatric Association, 1994). Defense levels differentiate one from another for their defensive function and level of adaptiveness, which are described in Table 1. Their assessment may inform about the most used defensive patterns, which reveal what defensive function is more frequently activated in response to internal conflicts or external stressors. For example, two individuals who use 40% of defenses belonging to the neurotic defensive category can have a very different defensive profile depending on whether they use a more obsessional or neurotic defense level. Similarly, high use of action and major image-distorting defense levels is very different from high use of disavowal and minor image-distorting defense levels, although they are all included in the immature defensive category. Furthermore, these differentiations among individuals’ defensive functioning are extremely evident when we look at the deepest level of investigation, the individual’s use of 30 individual defense mechanisms.

**TABLE 1:** The defensive function of the seven hierarchically ordered defense levels.

Level 7: High adaptive defenses	High adaptive defenses are the individual’s most adaptive ways of handling stressors and are often considered synonymous of positive coping. Internal or external stressors are fully perceived without distortion and the need to adapt to them is fully appropriated to oneself. The individual attempts to maximize the positive expression and gratification of his or her own motives, acknowledging limitations of the self and recurring to external sources of help when available.
Level 6: Obsessional defenses	Obsessional defenses protect the individual from the awareness of unacceptable or threatening feelings associated with an idea (e.g., wish, fear, experience, memory, or though) by keeping distance from emotions, while remaining aware of the idea itself. As a result, feelings (emotional component) are largely kept out of awareness and indirectly expressed throughout minimization, generalization, or a series of contradictory statements.
Level 5: Neurotic defenses	Neurotic defenses reflect the experience that awareness of a wish, thought, or motive is unacceptable or threatening and must be kept out of awareness. The individual can experience feelings associated to an internal conflict or external stressor as long as full awareness of the idea (cognitive component) is blocked and expressed indirectly by way of a series of anomalous clues. Neurotic defenses are the most protean of all defense mechanisms, in that there are a seemingly infinite variety of ways to give partial expression of repressed ideas.
Level 4: Minor image-distorting defenses	Minor image-distorting defenses protect the individual from experiences that affect one’s self esteem, such as failure, criticism, or disappointment that cause feelings of weakness, powerlessness, or shame. These defenses temporarily prop up self-esteem and strengthen self-image by using image-distortion to dismiss any threatening aspect of the stressor. These distortions are not all encompassing like those of the major image-distorting defenses. Nonetheless, they don’t actually improve adaptation to the stressors.
Level 3: Disavowal defenses and autistic fantasy	Disavowal defenses reflect the perception of the individual that some aspects of internal experience external reality are unacceptable. By refusing to acknowledge these aspects of experience, the individual justifies not appropriating a problem as his or her own. The individual can further misattribute the problem to another source or reason, further covering up internal reality. This results in a failure both to acknowledge one’s own role in the origins of a problem and to consider potential ways of handling the immediate problem, given the assertion that one has no such role.
Level 2: Major image-distorting defenses	Major image-distorting defenses protect the individual from intolerable anxiety when self or object representations of conflicting meaning are triggered. The individual keeps positive and negative representations separated and simplify the perception of self and others as either all good, powerful, and invulnerable or all bad, unworthy, powerless, and vulnerable. The individual then treats these distorted images in ways consistent with this perception. These defenses protect the self from the anxiety attending a sense of imminent threat of being punished, physically or psychologically abused, abandoned, or even killed. However, oversimplifying self or others and reacting accordingly produces the negative consequent that others withdraw or react negatively.
Level 1: Action defenses	Action defenses reflect the perception of the individual that the immediate source of stress or conflict is external and that the experience is intolerable. The individual’s perception overlooks the internal sources of the distress, such as personal unacceptability of or limitations in awareness of one’s own wishes, fears, and inhibitions. Unable to contain attendant distress, these defenses operate to engage, manipulate, or counterattack the apparent external source. These defenses lead the individual to impulsive action on the environment or oneself, thereby releasing tension, gratifying wishes, and/or avoiding fears. However, this is done without anticipating negative consequences.

*Extensive description of defense levels published in Perry (2014).*

Training individuals to rate defenses reliably is time consuming, as are making the ratings themselves, both of which limit the use of such ratings in clinical setting. While the DMRS is necessary for some types of research, we developed the DMRS-Q to meet the needs of a quicker, more user-friendly computerized tool for the assessment of defense mechanisms in clinical setting (Di Giuseppe et al., 2020b,c).

The present article aims to illustrate the DMRS-Q and its assessment and scoring methodology. We will provide the definition and function of 30 defense mechanisms as reported in the DMRS manual (Perry, 1990) and present the five DMRS-Q items corresponding to each defense mechanisms. Moreover, we will provide instructions for coding defenses with the DMRS-Q online software^1^ and syntax for the scoring. Finally, we will provide directions for data interpretations of the DMRS-Q qualitative and quantitative output.

## Methods

### Measure Development

Based on the DMRS definition and function, and discriminations from near-neighbor defenses, we developed a pool of 300 items – 10 statements for each defense mechanism – that refer to verbal and nonverbal expressions, distorted perceptions, personal mental states, relational dynamics, and way of coping that emerge on occasions when the subject experiences internal or external stress or conflict. A group of researchers trained on the DMRS was asked to indicate the five items for each defense mechanism that best captured a full range of manifestations according to the DMRS criteria. Following reviewers’ comments and basing on item’s clarity, simplicity, and non-redundancy, we selected the best five items for each defense mechanisms obtaining a final set of 150 items that constitute the DMRS-Q. We decided to select the DMRS-Q item pool, based on the coverage of manifestations of each DMRS defense, rather than on maximizing internal consistency of the items to overall defense score. This methodological approach was based on author’s hypothesis that reproducing the widely validated DMRS in an easy-to-use Q-sort version would guarantee strong psychometric properties because of the gold-standard theoretical background. Although we are aware that this is far from the usual methodological approach applied for the development of new psychometric tools, our preliminary analyses on validity and reliability of the DMRS-Q (Di Giuseppe et al., 2014; Békés et al., 2021; Tanzilli et al., 2021) confirmed our hypothesis on the importance of a strong theoretical base for a measure with statistically relevant properties.

### Definitions and Function of Defense Mechanisms and Defense Mechanisms Rating Scales Q-Sort Items

The DMRS-Q provides five items for each of the 30 defense mechanisms included in the hierarchy. A comprehensive overview of definitions, functions and DMRS-Q items is provided below. Tables 2–8 display DMRS-Q items for each defense included in each defense level; they are reported in descending order of defensive maturity. The following descriptions of the individual defenses are reproduced or adapted from the DMRS manual (Perry, 1990), with permission of the author, JP to provide the definitional basis for the DMRS-Q items in Tables 2–8.

**TABLE 2:** High-adaptive defense level: Definition, function and DMRS-Q items of defenses affiliation, altruism, anticipation, humor, self-assertion, self-observation, sublimation, and suppression.

Defense mechanism	DMRS-Q items
Affiliation	ITEM 22: Whenever the subject brings a personal problem to someone for help or advice, the subject is not expecting the other to take care of it, but rather to help come up with a solution which the subject will then implement. ITEM 25: The subject describes an important conflict or external stress in which affiliation played a major emotional role in coping as evident by the description of characteristics of the help received, the individuals or organization involved, and the sense that something was taken away from the experience. ITEM 44: When the subject describes seeking help from others, there is a sense of having learned something from the interchange. ITEM 66: When confronted with emotional conflict or stressful situations, the subject describes confiding in someone. Emotionally meaningful sharing led to enhancement of coping skills, or direct assistance beyond what the subject would have done alone. ITEM 93: When dealing with an emotionally difficult situation, the subject reports that talking to others helps the subject think through how best to handle the problem.
Altruism	ITEM 11: The subject helps others who are experiencing a problem they cannot adequately deal with alone. The problem appears to have a personal meaning to the subject related to similar experiences in the subject’s past (e.g., ‘It made me feel good to help someone in the same position that I once found so difficult.’). ITEM 15: The subject finds it personally rewarding to help others who are suffering. The subject participates in organizations or groups that help other people in direct person-to-person ways. In this context, the subject gives direct help to others, which the subject apparently finds rewarding. ITEM 104: The subject reacts to a difficult or dangerous situation for someone else by interposing him or herself to protect the other person. While not reckless, the subject may put him or herself at personal physical or material risk in doing so. ITEM 132: The subject helps others who are at a loss to cope with a problem or situation, possibly including standing up to authority. It is clear that the subject obtains some personal gratification or mastery from the meaning of helping, beyond any overt reward obtained.
Anticipation	ITEM 43: Ahead of an important performance or occasion, the subject practices imagining him or herself in the situation to be both better prepared and less anxious. ITEM 46: The subject describes small events in his or her life in which he or she characteristically mentions thinking about their outcomes ahead of time and emotionally preparing in some way for them. ITEM 62: In confronting a new situation or an unknown task, the subject tries ahead of time to be aware of the emotional challenges and plan for whatever resources that will aid and comfort the subject in the new situation. ITEM 65: The subject describes emotionally meaningful vignettes of upcoming stressful situations in which the subject fully prepared him or herself emotionally as to how to handle it. ITEM 78: In dealing with interpersonal conflicts, the subject tries to imagine how others might respond in planning how to deal with them, but without obsessing or over planning.
Humor	ITEM 18: The subject makes amusing or ironic comments about embarrassing situations to diffuse them. ITEM 37: The subject can make humorous remarks about him or herself or others without saying negative, hurtful, or deprecating things. ITEM 40: In confronting difficult situations which the subject cannot change, the subject uses humor about the situation to mitigate the negative feelings arising. ITEM 51: The subject diffuses a difficult situation with others by making a pertinent joke that centers on some important point that all can acknowledge without being at anyone’s expense, thereby fostering cooperation. ITEM 119: When confronted by a situation fraught with competitive, hostile, or jealous feelings, the subject reveals something about him or herself in a self-deprecatory, ironic, or amusing way to diffuse the tension.
Self-assertion	ITEM 23: When pursuing something desirable, including a relationship with someone, the subject can use his or her talents and charms to attract the other, without feeling ashamed or guilty if unsuccessful. ITEM 90: When the subject has a physical or emotional or practical problem, the subject takes steps to deal with his or her needs – possibly including initiating getting help – rather than ignore them or hope they will take care of themselves. ITEM 105: When someone is impolite, dismissive, or derogatory toward the subject, the subject can stand up for him or herself appropriately, even if the subject cannot change the other’s attitude or command an apology. ITEM 109: The subject can disagree with others and express opinions without being overly hostile, devaluing, or manipulative of others. ITEM 146: When confronted with emotionally difficult situations, the subject expresses his or her thoughts, wishes, or feelings clearly and directly without inhibition or excess.
Self-observation	ITEM 9: When talking with someone about a personally charged topic, the subject displays an accurate view of him or herself and can see how he or she appears from the other person’s point of view. ITEM 32: When confronting emotionally important problems, the subject can reflect upon relevant personal experiences and explore emotional reactions. This allows the subject to adjust better to limitations and compromises, possibly leading to more fulfilling outcomes. ITEM 58: In interpersonal conflicts, the subject uses an understanding of his or her reactions to facilitate understanding others’ points of view or subjective experiences. This may make the subject a better negotiator or collaborator. ITEM 77: When considering an emotionally important decision, the subject explores his or her own motives and limitations to arrive at a more fulfilling decision. ITEM 91: When the subject reflects on past experiences, he or she can relive distressing feelings and make connections between events and feelings and develop understanding thereby changing how the subject views the past and possibly similar situations in the present.
Sublimation	ITEM 14: In describing any personal artistic or creative activities – such as writing, music, art, or acting – the subject appears to transform emotional conflicts or unfulfilled wishes from elsewhere in life, helping to shape the creative activity or product. ITEM 36: The subject describes emotional conflictual situations in which some of the feelings or dissatisfaction are channeled into creative or artistic activities. The resulting creative products – such as a poem or painting – give the subject a sense of mastery or relief from the conflicts.
	ITEM 63: Whenever engaging in a creative activity, the subject finds the process of creation itself satisfying, apart from any satisfaction with the final product. ITEM 97: Following experiences of emotional distress or conflict, the subject engages in sports or other physical activities which are an invigorating outlet for any lingering frustrations. ITEM 100: Following some strong experiences, the subject engages in his or her ordinary activities but with less effort, greater accomplishment and more pleasure than they normally would require or yield.
Suppression	ITEM 49: When presented with an external demanding situation over which the subject has no control, the subject can accept the demand, putting negative feelings aside to deal with what must be done. ITEM 117: When the subject experiences a desire that if acted upon would have bad consequences, the subject is able to decide consciously to put the desire aside and not act upon it. ITEM 128: When the subject experiences a salient personal limitation or problem, rather than pretending it is not a problem, the subject acknowledges and accepts it, which allows the subject to avoid exacerbating problems. For example, acknowledging an addiction and accepting that one must avoid using the desired substance. ITEM 131: When attending to something emotionally important, if interrupted by something more urgent, the subject attends to the interruption as needed, but later returns and finishes dealing with what had to be postponed. ITEM 150: When presented with an emotionally charged situation, the subject can postpone dealing with his or her feelings to attend to the things that need to be done immediately. The feelings don’t get in the way or distract the subject, because the subject is able to give them adequate attention later.

**TABLE 3:** Obsessional defense level: Definition, function and DMRS-Q items of defenses isolation of affects, intellectualization and undoing.

Defense mechanism	DMRS-Q items
Isolation of affects	ITEM 28: When telling an emotionally meaningful story, the subject states that he or she does not have specific feelings that one would expect, although the subject recognizes that he or she should feel something. ITEM 31: In talking about a meaningful, emotionally charged experience, the subject talks in a detached way, as if he or she is not in touch with the feelings that should surround it. ITEM 39: The subject clearly describes the details of either positive or distressing or traumatic experiences but fails to show any attendant emotion in tone of voice, facial expression, or bodily expression. ITEM 107: The subject talks as if emotionally detached from whatever he says about himself or his experiences. ITEM 140: The subject describes events with good detail, but without mention of any attendant feelings, like a reporter describing the narrative of someone’s life, but devoid of personal reactions.
Intellectualization	ITEM 4: When confronting personal issues, the subject tends to ask general questions, as if getting general information or answers from others will elucidate his or her own feelings and concerns. As a result, personal reactions are kept at a distance. ITEM 26: The subject talks about his personal experiences by making general statements that appear accurate but somehow avoid revealing specific personal feelings and reactions. ITEM 53: There is a lifeless quality to most of the subject’s descriptions of his feelings and reactions, because the subject tries to explain them intellectually rather than experience or express them. For example: ‘My present predicament is an inevitable product of my parents’ extreme expectations and other parental experiences when growing up.’ ITEM 57: The subject distances him or herself from his or her own feelings by speaking about him or herself in the second or third person a lot, as if the subject were talking about someone else. ITEM 60: Whenever focusing on personal issues or experiences the subject tends to generalize or even discuss things in a logical or scientific way, thereby keeping his feelings and experiences very distant.
Undoing	ITEM 48: When another person tries to clarify a statement made by the subject, the subject says thing like ‘well, not really’ or ‘not exactly’ followed by qualifications that do not clearly clarify things. Because the subject is wary of committing him or herself to any statement, the listener may be unsure as to the subject’s definite opinion. ITEM 67: The subject spontaneously describes some of his or her actions which are followed by actions that are of the opposite intent, as if every action must be balanced by an equal but opposite action. The subject is aware of the contradiction which may seem vexing or ironic. ITEM 70: The subject prefaces a strong statement about a topic with a disclaimer, to the effect that what he or she is about to say may not be true. ITEM 81: The subject conveys opinions about something or someone with a series of opposite or contradictory statements, as if uncomfortable with taking a clear stand one way or the other. ITEM 83: After the subject has done something that probably results in a feeling of guilt or shame, the subject makes an act of reparation, as if sorry. However, the subject focuses on the act but avoids dealing with the sense of guilt or shame as one would whenever making a normal apology.

**TABLE 4:** Neurotic defense level: Definition, function and DMRS-Q items of defenses repression, dissociation, reaction formation, and displacement.

Defense mechanism	DMRS-Q items
Repression	ITEM 13: The subject keeps unpleasant things vague: he or she has trouble remembering or can’t recall specific examples, when at least some should be forthcoming. This may include loss of memory for whole periods of time (e.g., childhood). ITEM 47: At points when a topic is emotionally loaded, the subject forgets what he or she is talking about and seems to get lost while talking. ITEM 50: When discussing a topic that brings up negative, conflicting feelings, the subject prefers to keep things vague, reflected in very vague, general or inexact statements. ITEM 108: The subject cannot remember certain facts which would normally not be forgotten, such as a distressing incident, reflecting some uneasy feelings about the topic. ITEM 136: When certain feelings or wishes would arise, the subject gives some evidence of them – such as crying or appearing anxious but cannot clearly identify in words the specific feeling or the specific ideas that give the wish a clear meaning.
Dissociation	ITEM 8: The subject behaves or says something in a very uncharacteristic way that expresses an uninhibited impulse operating out of the subject’s usual control, yet the subject is surprised by it (e.g., “I threw a glass of water in my friend’s face, but I don’t know what made me do it’). ITEM 27: The individual describes fugue states, amnesia (not alcoholic blackouts), multiple personality, spontaneous trance states, or temporary loss of sensory or motor function. ITEM 30: In response to an emotionally charged situation, the subject suddenly becomes confused, depersonalized, “spaced out,” or can’t think or talk about the topic. Consciousness becomes clouded to a lesser or greater extent. ITEM 41: In response to a distressing topic or situation, the subject develops a symptom, such as headache, stomach pain, or loss of an ability to do something, which temporarily eclipses awareness of what was distressing. The symptom may have a symbolic relationship to the type of distress. ITEM 73: The subject associates with or is fascinated by people who do very uninhibited, dramatic, or socially outrageous things, which appear to express some of the subject’s own inhibited wishes. Nonetheless, the subject is unaware of any such connection.
Reaction formation	ITEM 52: When confronting a personal wish about which the subject may feel guilty, the subject does not acknowledge or express it, but substitutes an opposite attitude against the wish, for instance, a desire is supplanted by renunciation or anger at anything to do with the desire. ITEM 55: The subject is very compliant, agreeing to most everything the interviewer points out, when some disagreement and discussion would be expected. ITEM 74: In dealing with people who are angry or abusive, the subject is cooperative and nice and eager to please, failing to express any negative feelings which might be expected. ITEM 96: In relationships, the subject has an attitude of giving much more than he or she receives but is unaware of the imbalance. ITEM 99: In fearful situations, the subject does not show expected fear, but reacts with exaggerated enthusiasm or courage, failing to acknowledge the fear.
Displacement	ITEM 1: In dealing with an important problem that makes the anxious, the subject prefers to focus on minor or unrelated matters instead, which distracts the subject away from the central problem, for example, cleaning or organizing rather than working on projects that need to be done. ITEM 64: The subject directs strong feelings toward a person or object who has little to do with the subject but who may bear similarities to someone significant to the subject. The subject may be somewhat puzzled by the ‘reason’ for the strength of these feelings. ITEM 69: When confronting emotionally charged topics, the subject tends not to address concerns directly and fully but wanders off to tangentially related topics that are emotionally easier for the subject to discuss or prefers to pay attention to someone else dealing with a similar situation. This can include preferring to read or watch a film portraying people dealing with similar problems. ITEM 122: When discussing an affect-laden event, the subject expresses more feelings directed toward incidental details or issues than about the major point or effect of the event, perhaps appearing “picky.” ITEM 125: The subject gets irritated easily by minor things that bother him or her and tends to lose a focus on the main things that need attention.

**TABLE 5:** Minor image-distorting defense level: Definition, function and DMRS-Q items of defenses devaluation of Self-image, devaluation of other’s image, idealization of self-image, idealization of other’s image, and omnipotence.

Defense mechanism	DMRS-Q items
Devaluation of self-image	ITEM 12: The subject says demeaning things about him – whether somewhat funny or not – such as “I am so-ooooo stupid.” ITEM 29: The subject makes a lot of unwarranted negative, sarcastic, or biting statements about the self, but the individual can acknowledge some of their positive aspects, if these are pointed out. ITEM 34: When experiencing failure, disappointment, shame or loss of self-esteem, the subject dismisses the issue by saying something negative about him or herself, then dismisses the problem by moving to another topic and avoids focusing on the feelings. ITEM 56: The subject is preoccupied with real or exaggerated faults in him or herself, although he or she can acknowledge some realistic positive aspects, if these are pointed out. ITEM 147: When confronted by a personal disappointment the subject makes negative comments about him or herself but then avoids further discussion of the disappointment in any detail.
Devaluation of other’s image	ITEM 54: When a topic brings with it feelings of disappointment, shame or loss of self-esteem, the subject dismisses the issue by finding some fault or criticism elsewhere or by uttering obscene comments about it. ITEM 82: The subject devalues others’ accomplishments or motives, to minimize their significance, but he or she quickly dismisses such topics rather than dwell on them. ITEM 85: When asked to discuss something about him or herself, the subject diverts the focus to saying negative things about others, as if devaluing others will raise his or her own self-esteem. ITEM 111: The subject has negative things to say about a lot of individuals or objects, although he or she can acknowledge some of their positive aspects, if these are pointed out. ITEM 143: The subject makes sarcastic or biting statements about others to minimize their positive qualities and dismiss any competition or threat they may pose.
Idealization of self-image	ITEM 38: When confronted with any negative aspects of him or herself, the subject appears to downplay or ignore them by substituting talk about positive self-attributes instead. ITEM 71: The subject makes many references to how important he or she is with an emphasis on self-image, rather than real accomplishments which might make the person important to others. ITEM 87: The subject tells stories in which others are saying positive things about him or herself. ITEM 133: The subject takes pleasure in referring a lot to his or her own positive but superficial attributes, like being beautiful, lovable, smart, well-dressed, worthy, a center of attention. This may be true even if the subject longs for qualities that are only imagined, wished for, or in the past. ITEM 135: When confronted with problems, the subject prefers to dwell on his or her own positive qualities, such as being lovable, smart, beautiful, creative, “the best,” as if those qualities will take care of the problems.
Idealization of other’s image	ITEM 16: The subject makes many references to how important certain people or objects are with an emphasis on their image, rather than real abilities or accomplishments which might make the person or object important to others. ITEM 17: The subject tells stories in which he or she says glowing positive things about another person or object, without giving much detail to back it up. ITEM 95: When confronted with problems, the subject prefers to dwell on the positive qualities of others on whom he or she relies, such as being lovable, smart, beautiful, creative, “the best,” as if those qualities will take care of the problems. ITEM 138: The subject takes pleasure in referring a lot to positive but superficial attributes of others, like being beautiful, lovable, smart, well-dressed, worthy, a center of attention. This may be true even if the subject longs for qualities that are only imagined, wished for, or in the past. ITEM 139: When confronted with any negative aspects of others important to the subject, the subject appears to downplay or ignore them, by substituting talk about the positive image or attributes instead.
Omnipotence	ITEM 7: The subject talks about how capable he or she is of influencing events or famous and important people. However, the emphasis is on the sense of personal power or abilities, rather than the detailed stories that support the claims as real. ITEM 10: The subject acts in a very self-assured way and asserts an ‘I can handle anything’ attitude, in the face of problems that he or she in fact cannot fully control. ITEM 68: The subject makes clearly false statements about his own special powers and abilities (these may or may not be delusional). ITEM 126: There is excessive bravado in discussing problems or personal accomplishments that stands out as excessive or unrealistic. ITEM 129: The subject is very grandiose in describing personal plans, accomplishments or abilities, perhaps comparing him or herself to famous people.

**TABLE 6:** Disavowal defense level: Definition, function and DMRS-Q items of defenses denial, rationalization, projection, and autistic fantasy.

Defense mechanism	DMRS-Q items
Denial	ITEM 20: When confronted with topics that might be personally meaningful, the subject denies they are important and refuses to talk about them further. ITEM 33: Contrary to the evidence from the interview, the subject claims to have done something that in all likelihood he or she did not do, and may become irritated if confronted with any discrepancy. ITEM 121: Whenever talking about potentially distressing events or experiences, the subject strongly claims not to have any feelings about the topic, although this seems highly unlikely. ITEM 124: Whenever asked about things the subject did or felt, the subject denies any involvement, does not want to talk about them or avoids explaining his or her reluctance. ITEM 137: The subject is hard to talk with, responding to many questions with answers like “no” or “not really” and does not elaborate, rather than giving some fuller answers which one would normally expect.
Rationalization	ITEM 19: To avoid taking responsibility for one’s actions or misdeeds, the subject makes excuses or points out others’ contributions to the problem, thereby minimizing his or her own role. ITEM 42: The subject avoids feelings of guilt or shame by justifying his actions or by referring to external reasons that impelled him to act. ITEM 59: When discussing a problem that the subject contributed to, the subject explains his or her own actions far more than necessary, as if explaining away his or her own fault. ITEM 86: Whenever confronted about his or her own feelings or intentions, the subject avoids acknowledging them by giving a plausible explanation that covers up the real subjective reasons. ITEM 120: Whenever discussing something uncomfortable about him or herself, the subject tries to convince someone else of a more positive explanation, as if lying to him or herself about the truth.
Projection	ITEM 112: When others comment or inquire about the subject’s own feelings, actions, or intentions, the subject is very elusive or frankly denies the material, but the subject subsequently talks about similar feelings, actions, intentions, etc., in others. ITEM 115: When experiencing or confronted with a problem, the subject shames, humiliates, or blames someone else for the problem, ignoring his or her own role. ITEM 123: An attitude of suspiciousness or prejudice toward a group of other individuals, allows the subject not to express an interest in the same motives or feelings but remain blind to them in him or herself. ITEM 134: When others ask the subject questions, the subject is suspicious about others’ real reasons or motives for the question. ITEM 141: The subject perceives others as untrustworthy, unfaithful, or manipulative when there is no objective basis for these concerns. This may even appear paranoid.
Autistic fantasy	ITEM 2: The subject has repetitive or serial daydreams to which he or she retreats in lieu of real life social relationships. ITEM 24: The subject daydreams a lot, not in a way that leads to creative planning or action, but simply for its own gratification, in lieu of action. ITEM 106: In dealing with some problems, the subject prefers to daydream about solutions, as a substitute for planning direct, realistic, and effective actions. ITEM 110: Whenever being self-assertive would be helpful, the subject may act passively but later withdraw into fantasies of being assertive or aggressive toward others as a compensation. ITEM 148: The subject gets intensely involved in fantasy roles or actions that express wishes and feelings that the subject does not express in real life. For example, living out a role in a social situation or game or which has no connection to real life ways in which the subject expresses him or herself.

**TABLE 7:** Major image-distorting defense level: Definition, function and DMRS-Q items of defenses splitting of self-image, splitting of other’s image, and projective identification.

Defense mechanism>	DMRS-Q items>
Splitting of self-image	ITEM 3: The subject has periods of saying highly positive things about him or herself, and other periods saying highly negative things about him or herself, without appearing to notice the contradiction and without addressing it, other than to feel confused about him or herself at moments. ITEM 6: The subject speaks of him or herself in a wholly negative way at times, as if there is nothing positive or redeeming about him or herself. ITEM 98: The subject expresses a series of highly unrealistic positive attributes about him or herself whereas at another point the subject sees only negatives in him or herself. The subject dismisses attempts to see things in a balanced more realistic way. ITEM 142: The subject tends to highlight objects with an emotional meaning that matches his or her own emotional tone at the moment. Any feeling that doesn’t match this is ignored or denied. ITEM 145: Whenever saying something negative about him or herself, the subject rejects others’ attempts to explore positive or more balanced views, and paradoxically becomes even more confirmed in his or her own worthlessness.
Splitting of other’s image	ITEM 35: The subject experiences other people and objects in “black or white” terms, failing to form more realistic views that balance positive and negative aspects of them. ITEM 61: The subject attributes unrealistic positive characteristics to an object, such as being all-powerful, omni-benevolent, a savior. Because of the unrealistic belief that the positive object will take care of one’s problems, the subject ignores the need to take care of some of his or her own needs. ITEM 92: The subject attributes unrealistic negative characteristics to an object, such as being all-powerful, malevolent, threatening. As a result, he or she makes some effort to protect him or herself from its influence, even though this response appears unwarranted or exaggerated. ITEM 94: The subject fails to recognize that someone may be untrustworthy, hurtful, or manipulative and does not draw obvious conclusions based on their behavior. This generally results in using very poor judgment about how others will treat the subject. ITEM 114: The subject expresses hatred toward someone or something and refuses to acknowledge anything that does not confirm the hatred.
Projective identification	ITEM 72: Sometimes the subject gets angry or fearful toward someone for no apparent reason, but then accuses the other person of intending to make him or her feel that way. ITEM 75: At times the subject’s feelings merge with those of another person and the subject assumes the other’s feelings and needs are exactly the same as the subject’s own. He or she then tends to “put words in the other’s mouth.” ITEM 101: In conversations, the subject sometimes seems confused about distinguishing his or her own feelings from those of the other person. ITEM 103: When the subject gets upset at someone, he or she gets very angry and loses control, but then blames the other person for making him or her lose control. Nonetheless, the subject may feel some guilt for losing control. ITEM 113: The subject feels provoked by someone when no obvious provocation is apparent. As the subject becomes angry, accusatory or verbally abusive, the subject provokes the same negative feelings in the other which the subject mistakenly believed the other person had at the outset.

**TABLE 8:** Action defense level: Definition, function and DMRS-Q items of defenses acting out, help-rejecting complaining, and passive aggression.

Defense mechanism>	DMRS-Q items>
Passive aggression	ITEM 45: At times when expressing an opinion or wish might be helpful, the subject fails to express himself adequately, instead finding indirect, even annoying ways to show his or her opposition to the influence of others, for example, being silent. ITEM 88: The subject fails to stand up for his or her interests and seems to let bad things happen to him or herself that could be prevented, maybe even assuming a “martyr” role. ITEM 89: While outwardly cooperative or compliant, the individual procrastinates and refuses to do things on time or as asked, even when it would be easy to do so. ITEM 102: When angry toward someone significant, the subject takes anger out on himself instead of expressing it directly. ITEM 116: The subject has “a chip on his or her shoulder” or a grudge, and seems to find reasons to feel unfairly treated, even when he or she is not.
Help-rejecting complaining	ITEM 21: The subject complains spontaneously about how others don’t really care, or have made his or her problems worse, even when there is clear evidence that others have tried to help. ITEM 84: The subject recites a litany of issues and problems but does not appear to be engaged in solving them, but rather prefers to complain. ITEM 127: The subject tends to exaggerate his or her complaints about a life problem or somatic symptom, making them seem worse or more significant than they are. ITEM 130: The subject complains about life issues or problems as if each were insoluble, and systematically rejects others’ suggestions about ways of handling them. ITEM 149: When the subject brings up a problem to discuss, others try to address the problem, but in response the subject skips to a different problem, thereby dismissing rather than engaging others in any suggestions offered.
Acting out	ITEM 5: The subject loses his or her temper easily. ITEM 76: In response to interpersonal disappointment or disagreement the subject tends to act impulsively, without reflection or considering the negative consequences. ITEM 80: The subject is often inhibited from expressing him or herself, but sometimes acts in uncontrolled ways to get or do something he or she wants, ignoring normal constraints. ITEM 118: Whenever the subject feels angry, disappointed or rejected by someone, the subject resorts to uncontrolled behaviors as an escape from distressing feelings, such as binge-eating, drinking, sexual escapades, drug use, reckless driving, or getting into trouble. ITEM 144: The subject tends to express feelings, wishes or impulses directly in behavior, not only words, without prior thought. However, afterward, he or she may feel guilty or expect some punishment.

#### High-Adaptive Defense Level: Affiliation

##### Definition

The individual deals with emotional conflicts, or internal or external stressors, by turning to others for help or support. By affiliating with others, the individual can express him or herself, confide problems, and feel less alone or isolated with a conflict or problem. This may also result in receiving advice or concrete help from the “auxiliary ego” that improves the individual’s ability to cope. Confiding leads to an increase in the individual’s coping capacity as the other individual supplies emotional validation and support. Affiliation does not include trying to make someone else responsible for dealing with one’s own problems, nor does it imply coercing someone to help, or acting helpless to elicit help. Affiliation is not shown simply by belonging to an organization (e.g., church, social club, Alcoholics Anonymous) or by seeing a counselor or therapist. Rather it is demonstrated by the give and take around conflicts and problems that occurs in the context of belonging to the organization, or by the confiding with others.

##### Function

Affiliation allies the individual’s emotional attachment needs with the wish to cope effectively with internal conflict or external stressors. The ability to cope is enhanced by seeking support from others, while attachment needs are also satisfied. Others may enhance the individual’s repertoire of ego skills by help with advice, modeling, planning, judgment, role playing, practicing, etc. Usually this is accompanied by a reduction in subjective tension achieved through expressing one’s feelings and sharing one’s conflicts.

#### High-Adaptive Defense Level: Altruism

##### Definition

The individual deals with emotional conflicts, or internal or external stressors, by dedication to fulfilling the needs of others, in part as a way of fulfilling his or her own needs. By using altruism, the individual receives some partial gratification either vicariously or as a response from others. The subject is usually aware to some extent that his or her own needs or feelings underlie altruistic actions. There may also be a direct reward or overt self-interested reason for the subject’s altruistic actions. To rate altruism present, there must be a clear, demonstrable, functional relationship between the individual’s feelings and the altruistic response.

##### Function

Altruism gratifies social and attachment needs while dealing with emotional conflict through helping others. In many cases, the conflict revolves around distress over past examples of confronting stressful situations for which one needed help that was somehow unavailable or insufficient. Altruism channels affects, such as anger, and experiences, such as powerlessness, into socially helpful responses that also enhance the individual’s sense of mastery over the past.

#### High-Adaptive Defense Level: Anticipation

##### Definition

The individual mitigates emotional conflicts, or internal or external stressors, by not only considering realistic, alternative solutions and anticipating emotional reactions to future problems, but experiencing the future distress by mentally bringing the distressing ideas and affects together. This rehearsal allows the individual to prepare a better adaptive response to the anticipated conflict or stressor.

##### Function

Using anticipation allows the individual to mitigate the effects of future stressors or conflicts. It requires being able to tolerate the anxiety attendant to imagining how a future situation may be distressing. By affective rehearsal (e.g., ‘how will I feel when this occurs?’) and planning future responses, the subject decreases distressing aspects of the future stressor. Anticipation also increases the likelihood of positive external outcomes and more positive emotional responses.

#### High-Adaptive Defense Level: Humor

##### Definition

The individual deals with emotional conflicts, or internal or external stressors, by emphasizing the amusing or ironic aspects of the conflict or stressor. Humor tends to relieve the tension around conflict in a way that allows everyone to share in it, rather than being at one person’s expense, as in derisive or cutting remarks. An element of self-observation or truth is often involved.

##### Function

Humor allows some expression of affects and wishes that are involved with conflict or stressor. Whenever conflict or external stressors block full expression of the affects or satisfaction of wishes, humor allows some symbolic expression of them and of the source of the conflict. The frustration emanating from the conflict is transiently relieved in a way that both self and others can smile or laugh at. This is especially evident around issues of the human condition in which certain stressors are inescapable.

#### High-Adaptive Defense Level: Self-Assertion

##### Definition

The individual deals with emotional conflicts, or internal or external stressors, by expressing one’s feelings and thoughts directly to achieve goals. Self-assertion is not coercive or indirect and manipulative. The goal or purpose of the self-assertive behavior is usually made clear to all parties affected by it.

##### Function

Self-assertion deals with emotional conflict through the direct expression of one’s feelings or wishes, and thereby relieves the anxiety or distress that occurs whenever internal or external countervailing forces prevent expression. Self-assertion does not require that the individual get his or her own way to be successful as a defense or adaptive response. Rather, it is also emotionally useful because it allows the individual to function (1) without the anxiety or tension that builds whenever feelings and wishes are unexpressed and (2) without a sense of shame or guilt for not speaking up for oneself in emotionally conflictual situations. The emotional consequences are worse when self-assertion is blocked by internal prohibitions, rather than by external factors alone, such as by a domineering person in authority.

#### High-Adaptive Defense Level: Self-Observation

##### Definition

The individual deals with emotional conflicts, or internal or external stressors, by reflecting on his or her own thoughts, feelings, motivation, and behavior. The person can “see himself as others see him” in interpersonal situations, and as a result is better able to understand other people’s reactions to him or her. The defense is not synonymous with simply making observations or talking about oneself.

##### Function

This defense allows the person to make the best adaptation to the demands of external reality based on having an accurate view of one’s own affects, wishes and impulses, and behavior. While self-observation does not change one *per se*, it is a precursor for seeking better adaptations of internal states to external reality. This defense allows the individual to grow and adapt better as he or she deals with stress.

#### High-Adaptive Defense Level: Sublimation

##### Definition

The individual deals with emotional conflicts, or internal or external stressors, by channeling rather than inhibiting potentially maladaptive feelings or impulses into socially acceptable behavior. This defense is to be rated present only when a strong functional relationship can be demonstrated between the feelings and response pattern. Classic examples of the use of sublimation are sports and games used to channel angry impulses, or artistic creation that expresses conflicted feelings.

##### Function

Sublimation allows the expression of wishes, impulses, or affects that the subject voluntarily inhibits because of their potentially negative social repercussions. The subject channels them instead into socially acceptable expression. The original aims and objects of the impulses, wishes, and affects are often modified considerably, resulting in a creative activity or product. For example, a hostile-competitive urge may be channeled into competitive sports or work, or sexual impulses may be expressed through creative dance or art. The result of sublimation is that the original impulses, etc. are allowed some expression while the resulting activity or product may also bring some positive social approval or reward.

#### High-Adaptive Defense Level: Suppression

##### Definition

The individual deals with emotional conflicts, or internal or external stressors, by voluntarily avoiding thinking about disturbing problems, wishes, feelings, or experiences temporarily. This may entail putting things out of one’s mind until the right time to deal with them: it is postponing not procrastinating. Suppression may also entail avoiding thinking about something at the time because it would distract from engaging in another activity which one must do (e.g., not dwelling on tangential problems in order to deal with one pressing problem). The individual can call the suppressed material back to conscious attention readily, since it is not forgotten.

##### Function

Suppression keeps both the idea and affect associated with a stressor out of awareness in the service of attending to something else; however, suppressed material may be voluntarily brought back into full awareness. Distressing feelings are acknowledged but dealing with them is postponed until the subject feels more able or the timing is more appropriate. Neurotic anxiety is minimized, since the material is not repressed, although anticipatory anxiety may still be present until the stressor is dealt with.

#### Obsessional Defense Level: Isolation of Affects

##### Definition

The individual deals with emotional conflicts, or internal or external stressors, by being unable to experience simultaneously the cognitive and affective components of an experience, because the affect is kept from consciousness. In the defense of isolation, the subject loses touch with the feelings associated with a given idea (e.g., a traumatic event) while remaining aware of the cognitive elements of it (e.g., descriptive details). Only the affect is lost or detached while the idea is conscious. It is the converse of repression, where the affect is retained but the idea is detached and unrecognized. Sometimes the affect can be detached temporarily from its associated idea. The affect is felt later without association to the original experience and idea. Instead, there is an intervening neutral interval between cognizance of the idea and experience of the associated affects.

##### Function

Individuals who feel threatened by or anxious over the conscious experience of feelings can still deal with the related ideas and events comfortably when their associated affects are separated and kept out of awareness. Very often the isolated affects are associated with anxiety, shame, or guilt that would emerge if experienced directly. The tradeoff for avoiding the associated anxiety, shame, or guilt is that the individual misses out on experiencing the feelings in a way that adds evaluative information and which may be useful in making choices.

#### Obsessional Defense Level: Intellectualization

##### Definition

The individual deals with emotional conflicts, or internal or external stressors, by the excessive use of abstract thinking to avoid disturbing feelings.

##### Function

Intellectualization is a defense against affects or impulses in which the idea representing the affect or impulse is kept conscious and expressed as a generalization, thereby detaching or distancing the subject from the affect or impulse itself. The felt quality of emotions is lost, as is the urge in any impulse. The cognitive elements remain conscious, although in generalized or impersonal terms. The subject commonly refers to his or her experience in general terms or in the second or third person. One does not have to be bright or intelligent to use intellectualization. It is simply a cognitive strategy for minimizing the felt importance of problems in one’s affective life. Like other defenses, it can sometimes be seen in those with intellectual disabilities and organic brain syndromes.

#### Obsessional Defense Level: Undoing

##### Definition

The individual deals with emotional conflicts, or internal or external stressors, by behavior designed to symbolically make amends for negate previous thoughts, feelings, or actions.

##### Function

In this defense the subject expresses an affect, impulse or commits an action which elicits guilt feelings or anxiety. He or she then minimizes the distress by expressing the opposite effect, impulse, or action. The act of reparation then removes the individual from experiencing the conflict. In conversation the subject’s statements are immediately followed by qualifications bearing the opposite meaning from the original statement. To the observer this coupling of statement with contradictory statement may make it difficult to see what the subject’s primary feeling or intention really is. Misdeeds may be followed by acts of reparation to the intended object of the misdeed. The subject appears compelled to erase or undo his or her original action.

#### Neurotic Defense Level: Repression

##### Definition

The individual deals with emotional conflicts, or internal or external stressors, by being unable to remember or be cognitively aware of disturbing wishes, feelings, thoughts or experiences.

##### Function

Repression is a defense that protects the subject from being aware of what he is experiencing or has experienced in the past. The subject may experience a particular affect, impulse, or desire, but the actual awareness of what it is, that is, the idea associated with it, remains out of awareness. While the emotional elements are clearly present and experienced, the cognitive elements remain outside of consciousness.

#### Neurotic Defense Level: Dissociation

##### Definition

The individual deal with emotional conflicts, or internal or external stressors, by a temporary alteration in the integrative functions of consciousness or identity. In the defense of dissociation, a particular affect or impulse which the subject is not aware of operates in the subject’s life out of normal awareness. Both the idea and associated affect or impulse remain out of awareness but are expressed by an alteration in consciousness. While the subject may be dimly aware that something unusual takes place at such times, full acknowledgment that his or her own affect or impulses are being expressed is not made. Dissociation may result in a loss of function or in uncharacteristic behavior.

##### Function

Dissociated material is commonly experienced as too threatening, too conflict-laden, or too anxiety-provoking to be allowed into awareness and fully acknowledged by the subject. Examples of common threatening material include recollection of a trauma with attendant fear of death and feelings of powerlessness, or a sudden impulse to kill an intimate associate. Dissociation allows expression of the affect or impulse by altering consciousness which allows the individual to feel less guilty or threatened.

#### Neurotic Defense Level: Reaction Formation

##### Definition

The individual deals with emotional conflicts, or internal or external stressors, by substituting behavior, thoughts, or feelings that are diametrically opposed to his or her unacceptable thoughts or feelings.

##### Function

In reaction formation an original impulse or affect is deemed unacceptable by the subject and an unconscious substitution is made. Feelings, impulses, and behaviors of opposite emotional tone are substituted for the original ones. The observer does not see the alteration, *per se*, but only the end product. By supplanting the original unacceptable feelings by its opposite, the subject avoids feelings of guilt. In addition, the substitution may gratify a wish to feel morally superior. Reaction formation is reasonably inferred when a subject reacts to an event with an emotion opposite in tone to the usual feelings evoked in people.

#### Neurotic Defense Level: Displacement

##### Definition

The individual deals with emotional conflicts, or internal or external stressors, by generalizing or redirecting a feeling about or a response to an object onto another, usually less threatening, object. The person using displacement may or may not be aware that the affect or impulse expressed toward the displaced object was really meant for someone else.

##### Function

Displacement allows the expression of an affect, impulse, or action toward a person or other object with some similarity to the actual object which initially aroused the affect or impulse. The affect or impulse is fully expressed and acknowledged but is misdirected to a less conflictual target. Displacement allows more expression and gratification, albeit toward the wrong targets, than other neurotic level defenses.

#### Minor Image-Distorting Defense Level: Devaluation

##### Definition

The individual deals with emotional conflicts or internal or external stressors by attributing exaggeratedly negative qualities to oneself or others.

##### Function

Devaluation refers to the use of derogatory, sarcastic, or other negative statements about oneself or others to boost self-esteem. Devaluation may fend off awareness of wishes or the disappointment when wishes go unfulfilled. The negative comments about others usually cover up a certain sense of vulnerability, shame or worthlessness which the subject experiences vis a vis expressing his own wishes and meeting his own needs.

#### Minor Image-Distorting Defense Level: Idealization

##### Definition

The individual deals with emotional conflicts, or internal or external stressors, by attributing exaggerated positive qualities to self or others.

##### Function

In the defense of idealization, the subject describes real or alleged relationships to others (including institutions, belief systems, etc.) who are powerful, revered, important, etc. This usually serves as a source of gratification as well as protection from feelings of powerlessness, unimportance, worthlessness, and the like. The defense accomplishes a sort of alchemy of worthiness by association. The subject believes certain others to be good and powerful in an exaggerated way and while able to acknowledge factual aspects of any faults or shortcomings in the idealized person, they dismiss their significance, thereby preserving a sterling image of the person, or object.

#### Minor Image-Distorting Defense Level: Omnipotence

##### Definition

Omnipotence is a defense in which the subject responds to emotional conflict or internal and external stressors by acting superior to others, as if one possessed special powers or abilities.

##### Function

This defense commonly protects the subject from a loss of self-esteem that is a consequence whenever stressors trigger feelings of disappointment, powerlessness, worthlessness, and the like. Omnipotence subjectively minimizes the latter experiences, although they may remain objectively obvious to others. Self-esteem is artificially propped up at the expense of positively distorting one’s self-evaluation in response to real experiences which bring up contrary feelings.

#### Disavowal Defense Level: Denial

##### Definition

The individual deals with emotional conflicts, or internal or external stressors, by refusing to acknowledge some aspect of external reality or of his or her experience that would be apparent to others. The subject actively denies that a feeling, behavioral response, or intention (regarding the past or present) was or is not present, even though its presence is considered more than likely by the observer. The subject is blinded to both the ideational and emotional content of what is denied. This excludes ‘psychotic denial” in which the subject refuses to acknowledge a physical object or event within the subject’s field in the present time.

##### Function

Neurotic denial serves to prevent the subject who uses it and anyone querying him from recognizing specific feelings, wishes, intentions, or actions for which the subject might be responsible. The denial avoids admitting or becoming aware of a psychic fact (idea and feeling) which the subject believes would bring him aversive consequences (such as shame, grief, or other painful affect). The evidence for this is clear whenever a subject breaks through his own denial and experiences shame or other emotion at what he learns about himself, often apologizing to the interviewer and so forth.

#### Disavowal Defense Level: Rationalization

##### Definition

The individual deals with emotional conflicts, or internal or external stressors, by devising reassuring or self-serving but incorrect explanations for his or her own or others’ behavior.

##### Function

Rationalization involves the substitution of a plausible reason for a given action or impulse on the subject’s part, when a motive that is more self-serving or difficult to acknowledge is evident to the outsider. While the underlying covert motivation may be selfish, it may also involve caring or loving feelings which the subject finds uncomfortable. The subject is usually thought to be unaware or minimally aware of his true underlying motive; instead, he or she sees only the substituted, more socially acceptable reason for the action. The subject’s reasons commonly have nothing to do with any personal satisfaction, and thus disguise his or her real impulse or motive, although any related affect may still show.

#### Disavowal Defense Level: Projection

##### Definition

The individual deals with emotional conflicts, or internal or external stressors, by falsely attributing his or her own unacknowledged feelings, impulses, or thought to others. The subject disavows his or her own feelings, intentions, or experience by means of attributing them to others, usually by whom the subject feels threatened and to whom the subject feels some affinity.

##### Function

Non-delusional projection allows the subject to deal with emotions and motives which make him feel too vulnerable (especially to shame or humiliation) to admit having himself. Instead he concerns himself with these same emotions and motives in others. The use of projection therefore commits the subject to a continual concern with those on whom he has projected his inner feelings as a way to minimize awareness of them himself.

#### Disavowal Defense Level: Autistic (or Schizoid) Fantasy

##### Definition

The individual deals with emotional conflicts, or internal or external stressors, by excessive daydreaming as a substitute for human relationships, more direct and effective action, or problem solving. Fantasy denotes the use of daydreaming as either a substitute for dealing with or solving external problems or as a way of expressing and satisfying one’s feelings and desires. While the subject may be aware of the ‘I’m just pretending’ quality of the fantasy, nonetheless, it may be the closest that he or she ever comes to expressing or gratifying the need for satisfying interpersonal relationships.

##### Function

Fantasy allows the subject to obtain some temporary, vicarious gratification by daydreaming a solution to a real-world problem of conflict. The subject feels good while using fantasy and momentarily bypasses the conviction of powerlessness. In fact, during fantasy the opposite conviction (i.e., grandiosity) may be in operation, that one can do anything. Fantasy is maladaptive only when it short-circuits rather than rehearses attempts to deal with the real world by substituting dream world gratification. Sometimes, there may be a wholesale substitution of daydream activity in the place of real world attempts to meet needs and solve conflicts. This occurs without any loss of the ability to perceive and test external reality. The subject knows the difference between reality and fantasy life.

#### Major Image-Distorting Defense Level: Splitting

##### Definition

The individual deals with emotional conflicts, or internal or external stressors, by viewing himself or herself or others as all good or all bad, failing to integrate the positive and negative qualities of the self and others into cohesive images; often the same individual will be alternately idealized and devalued. Splitting of self-images often occurs alongside splitting of others’ images, since they both were learned in response to the unpredictability of one’s early significant others. In splitting of self-images, the subject demonstrates that he has contradictory views, expectations, and feelings about himself which he cannot reconcile into one coherent whole.

The self-images are divided into polar opposites: at a given time the subject’s awareness is limited to those aspects of the self-having the same emotional feeling tone. He sees himself in “black or white” terms. At one point in time the subject believes he himself has good attributes, such as being loving, powerful, worthy, or correct, and having good feelings, or he believes the opposite: that he is bad, hateful, angry, destructive, weak, powerless, worthless, or always wrong and has only negative feelings about himself. The subject cannot experience himself as a more realistic mixture of both positive and negative attributes.

In splitting of other’s images (object images), the subject demonstrates that his views, expectations, and feelings about others are contradictory and that he cannot reconcile these differences to form realistic and coherent views of others. Object images are divided into polar opposites, such that the subject can only see one emotional aspect or side of the object at a time. Objects are experienced in black or white terms. Splitting is revealed in two major ways. The subject may initially describe an object wholly in one way but later describe that same object in opposite ways. Second, each object is simply lumped with other objects into good and bad, positive and negative camps. When the subject uses splitting of object images, he cannot integrate anything that doesn’t match his immediate experience of and feeling about a given object. All the attributes with the same feeling tone are highlighted, and contradictory views, expectations, or feelings about the object arc excluded from emotional awareness, although not necessarily from cognitive awareness.

##### Function

Splitting of object images and self-images is the subject’s defense against the anxiety of ruining the good images of people by allowing bad aspects of them to intrude upon the good. Splitting of self-images has one adaptive function: it minimizes the anxiety the subject would experience attempting to match his view of himself with how significant others will in fact see him and treat him. Instead, when seeing himself one way, the subject continues to see himself in the same valence no matter how others see him and treat him; contradictions then aren’t allowed into experience. This minimizes the disruptive, anxiety-provoking effects of trying to predict unpredictable people. The disadvantage is that the subject’s view of himself then becomes inflexible to the environmental realities, and the switch from good to bad views of himself is also unpredictable. This leaves the subject insensitive to more reasonable, predictable, and potentially more rewarding relationships outside of his original learning environment. In a better environment, the subject suffers from what was paradoxically so protective originally: an insensitivity to experiencing contradictory views of the self. Splitting of object images and self-images is the subject’s defense against the anxiety of ruining the good images of people by allowing bad aspects of them to intrude upon the good. Splitting of object images limits the anxiety the subject would feel in trying to discriminate how others will respond when he experiences or expresses his needs, feelings, etc. To see others as all good or all bad eliminates the anxiety-provoking task of trying to discern how others will behave toward the self, a task the subject believes to be impossible. Instead, the subject quickly categorizes people into good and bad camps based on subtle initial cues (e.g., ‘he frowned when I spoke, so he hates me”) or based largely on internal feeling states (e.g., “I feel so bad that I know you must hate me, so why should I open up to you?”). The defense is maladaptive, however, because the subject acts as unpredictably and irrationally toward others as he himself was treated; he forgoes the rewards he might attain if he were flexible in how he interacts with others. Using this defense, the subject wins some friends and makes some enemies, but not in a realistic way that considers the aggregate of others’ actual characteristics.

#### Major Image-Distorting Defense Level: Projective Identification

##### Definition

In projective identification the subject has an affect or impulse which he finds unacceptable and projects onto someone else, as if it was really that other person who originated the affect or impulse. However, the subject does not disavow what is projected – unlike in simple projection – but remains fully aware of the affects or impulses, and simply misattributes them as justifiable reactions to the other person! Hence, the subject eventually admits his affect or impulse, but believes it to be a reaction to those same feelings and impulses in others. The subject confuses the fact that it was he himself who originated the projected material. This defense is seen most clearly in a lengthy interchange in which the subject initially projects his feelings but later experiences his original feelings as reactions to the other. Paradoxically, the subject often arouses the very feelings in others he at first mistakenly believed to be there. It is then difficult to clarify who did what to whom first. This process is more extensive than simple projection, which involves the denial and subsequent external attribution of an impulse. Projective identification involves attribution of an image so that the whole object is seen and reacted to in a distorted light.

##### Function

Projective identification is the defense of the traumatized person who felt irrationally responsible for his or her traumas. The defense is called into play when interpersonal cues stimulate memories of traumatic situations or interchanges or their residues. The individual experiences the other person as doing something to him or herself that is threatening, which make him or her feel powerless. The subject reacts to this imagined (or partially real) threat by attacking and believing that his or her own actions are justified, despite provoking the other. Guilt over having aggressive wishes toward the other person emerges and is handled by identification with the other, reinforced ‘by the belief that the alleged threat attack on oneself is deserved. Paradoxically the subject often induces the very feeling of powerlessness and guilt in others that he or she feels, which may result in others backing away.

#### Action Defense Level: Passive Aggression

##### Definition

The individual deals with emotional conflicts, or internal or external stressors, by indirectly and unassertively expressing aggression toward others. There is a facade of overt compliance masking covert resistance toward others. Passive aggression is characterized by venting hostile or resentful feelings in an indirect, veiled, and unassertive manner toward others. Passive aggression often occurs in response to demands for independent action or performance by the subject or when someone has disappointed the subject’s wish or sense of entitlement to be taken care of, regardless of whether the subject has made this wish known. This term includes ‘turning against the self.’

##### Function

The person using passive-aggression has learned to expect punishment, frustration, or dismissal if he or she expresses needs or feelings directly to someone who has power or authority over him or her. The subject feels powerless and resentful. This expectation is most pronounced in hierarchical power relationships. Resentment is expressed by a passive stance: that the subject is entitled to the very things he doesn’t speak up for or that he is entitled to special dispensation. There is also some pleasure taken in the discomfort that the passive aggressive behavior causes others. Passive expression of anger through stubborn, inept, procrastinating, and forgetful behavior is quickly learned as a way to express: the conviction that the subject has the right to remain passive while expecting his needs to be met; to appear well-intentioned on the surface (overtly compliant), thus avoiding retaliation for the direct expression of affects, needs, or resentment; to express the resentment experienced toward those making demands by covert noncompliance that annoys others and obtain some satisfaction or vengeance, even if it means hurting oneself. In extremes, the resentment is not just expressed indirectly toward the other, but in fact, is turned 180 degrees around toward the self (turning against the self) to get at the other.

#### Action Defense Level: Help-Rejecting Complaining

##### Definition

Help-rejecting complaining (formerly called hypochondriasis, which term we do not us as it can be confused with the symptom disorder) involves the repetitious use of a complaint or series of complaint in which the subject ostensibly asks for help. However, covert feelings of hostility or resentment toward others are expressed simultaneously by the subject’s rejection of the suggestions, advice, or whatever others offer. The complaints may consist of either somatic concerns or life problems. Either type of complaint is followed by a ‘help-rejecting complainer’ response to whatever help is offered.

##### Function

Help-rejecting complaining is a defense against the anger the subject experiences whenever he or she feels the need for emotional reliance on others. The anger rises from the conviction, or often the experience that nobody will really satisfy the subject’s perceived needs. The subject expresses the anger as an indirect reproach by rejecting help as “not good enough” while continuing to ask for more of it. Instead of driving the other person away by the expression of anger, the use of help-rejecting complaining binds the person to the subject by the overt request for help. The subject’s expression of helplessness over the problem at hand reflects a sense of powerlessness to get the right help, comfort, and attention, while discharging resentment for the expected disappointment that enough help will not be forthcoming.

#### Action Defense Level: Acting Out

##### Definition

The individual deals with emotional conflicts, or internal or external stressors, by acting without reflection or apparent regard for negative consequences. Acting out involves the expression of feelings, wishes or impulses in uncontrolled behavior with apparent disregard for personal or social consequences. It usually occurs in response to interpersonal events with significant people in the subject’s life, such as parents, authority figures, friends, or lovers. This definition is broader than the original concept of acting out transference feelings or wishes during psychotherapy. It includes behavior arising both within and outside of the transference relationship. It is not synonymous with “bad behavior,” or with any symptom *per se*, although acting out often involves socially disruptive or self-destructive behavior. So-called acting out behaviors, such as physical fighting, or compulsive drug use, must show some relationship to affects or impulses that the person cannot tolerate to serve as evidence for the defense of acting out.

##### Function

Acting out allows the subject to discharge or express feelings and impulses rather than tolerate them and reflect on the painful events that stimulate them. The following elements are present. First, the subject has feelings or urges which he is inhibited from expressing. Experiencing the original impulse quickly results in a rise in tension and anxiety. Second, the individual bypasses awareness and ceases any attempt to delay, reflect upon, or plan a strategy to handle the impulse or feeling. Rather it is directly expressed in behavior without prior thought. This results in the expression of rather raw aggression, sex, attachment, or other impulses without taking the consequences into account. Following acting out, reflection may return, and the subject commonly feels guilty or expects some punishment, unless a further defense comes into play, such as denial or rationalization (“I was so angry, I had to do it. It was his fault for stirring me up.”). Acting out is maladaptive because it does not mitigate the effects of the internal conflict, and it often brings upon the subject serious, negative, external consequences.

### Coding Procedure

The DMRS-Q is a computer-based measure that can be used for clinical, research and teaching purposes by registering on the DMRS-Q platform (see text footnote 1 for registration and login). The software use is free of charge and provides the user with several functions, such as starting a new coding, revising previous ratings, downloading outputs and scoring sheets. At present the DMRS-Q is available in English and in Italian, although other languages may be added on the platform after appropriate validation.

**FIGURE 2:**
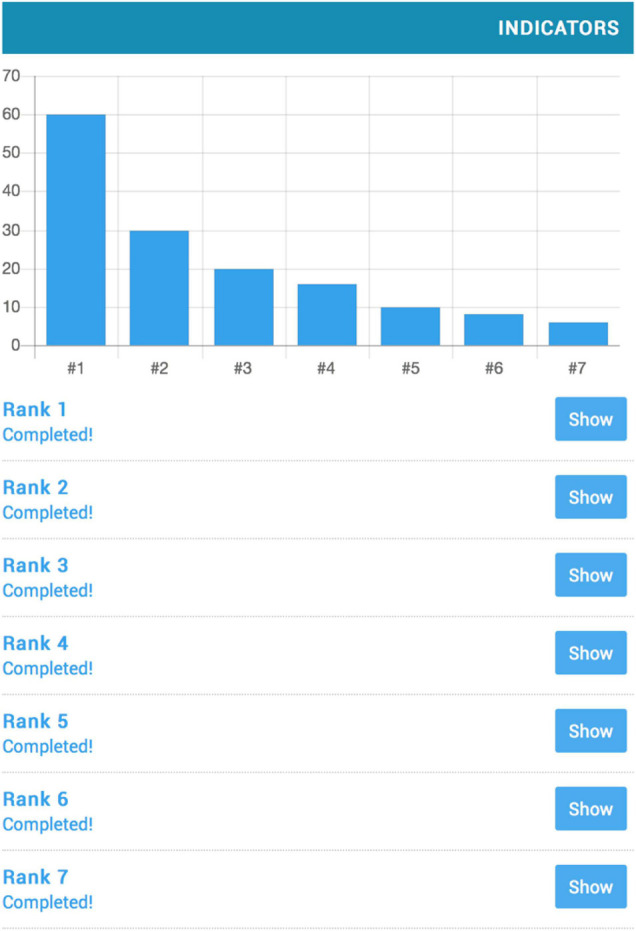
The DMRS-Q forced distribution (image extracted from the DMRS-Q web-app).

Like most Q-sort tools, the DMRS-Q coding procedure follows the rules of ranking items into a force distribution (Block, 1978; Brown, 1993, 1995). The 150 items must be ordered into seven ordinal ranks, corresponding to increasing level of descriptiveness, intensity or frequency. Higher ranks are less populated and include items that best describe the most characteristic defensive patterns activated by an individual. Conversely, lower ranks are more populated and include items that either do not apply or are only somewhat descriptive of the individual’s defensive profile. In ascending order of descriptiveness, DMRS-Q ranks are as follows: rank 1 (60 items) = not used at all; rank 2 (30 items) = very rarely used; rank 3 (20 items) = slightly or rarely used; rank 4 (16 items) = medium or sometimes used; rank 5 (10 items) = intensive or often used; rank 6 (8 items) = very intensive or frequently used; rank 7 (6 items) = almost always used. When all items are correctly ordered into the DMRS-Q forced distribution, as displayed in Figure 2, the rating is complete and ready to be sent for scoring output. For detailed directions of the DMRS-Q rating procedure a video-tutorial is available at https://www.youtube.com/watch?v=PP1ykSrGLkY&t=87s.

### Clinical Data and Training

Data required for a stable DMRS-Q rating might vary with the aim of its use. Coders must have sufficient information of the evaluated subject’s defensive functioning, directly observed or obtained from records. Since recorded and transcribed data are not essential, the DMRS-Q can be applied in multiple contexts. The required time for a DMRS-Q coding decreases depending on rater’s experience, ranging from about 60 min in the very first ratings to less than 15 min for expert coders. A 6-h training is highly suggested for reaching high reliability on all DMRS-Q quantitative scores, although a recent study demonstrated that untrained raters obtain acceptable to excellent reliability on most DMRS-Q scales (ICC ranging from 0.60 to 0.91) (Békés et al., 2021). In any case, for the correct use of the DMRS-Q it is essential to read the present manual for understanding the theoretical and methodological background behind the measure.

### Scoring System

The DMRS-Q scoring procedure is made with a software that extracts DPN and quantitative scores from the completed DMRS-Q rating. Formulas for quantitative scoring are displayed in Table 9.

**TABLE 9:** DMRS-Q quantitative scoring system.

	Items labels								
#	Defense	#	Defense	#	Defense	#	Defense	#	Defense
1	Displacement	31	Isolat_Affect	61	Splitting_Self	91	Self_Observat	121	Denial
2	Autis_Fantasy	32	Self_Observat	62	Anticipation	92	Splitting_Self	122	Displacement
3	Splitting_Other	33	Denial	63	Sublimation	93	Affiliation	123	Projection
4	Intellectualizat	34	Devaluat_Self	64	Displacement	94	Splitting_Self	124	Denial
5	Acting_Out	35	Splitting_Self	65	Anticipation	95	Idealizat_Other	125	Displacement
6	Splitting_Other	36	Sublimation	66	Affiliation	96	React_Format	126	Omnipotence
7	Omnipotence	37	Humor	67	Undoing	97	Sublimation	127	Help_Rej_Com
8	Dissociation	38	Idealizat_Self	68	Omnipotence	98	Splitting_Other	128	Suppression
9	Self_Observat	39	Isolat_Affect	69	Displacement	99	React_Format	129	Omnipotence
10	Omnipotence	40	Humor	70	Undoing	100	Sublimation	130	Help_Rej_Com
11	Altruism	41	Dissociation	71	Idealizat_Self	101	Proj_Identific	131	Suppression
12	Devaluat_Self	42	Rationalization	72	Proj_Identific	102	Passive_Aggr	132	Altruism
13	Repression	43	Anticipation	73	Dissociation	103	Proj_Identific	133	Idealizat_Self
14	Sublimation	44	Affiliation	74	React_Format	104	Altruism	134	Projection
15	Altruism	45	Passive_Aggr	75	Proj_Identific	105	Self_Assertion	135	Idealizat_Self
16	Idealizat_Other	46	Anticipation	76	Acting_Out	106	Autis_Fantasy	136	Repression
17	Idealizat_Other	47	Repression	77	Self_Observat	107	Isolat_Affect	137	Denial
18	Humor	48	Undoing	78	Anticipation	108	Repression	138	Idealizat_Other
19	Rationalization	49	Suppression	79	Altruism	109	Self_Assertion	139	Idealizat_Other
20	Denial	50	Repression	80	Acting_Out	110	Autis_Fantasy	140	Isolat_Affect
21	Help_Rej_Com	51	Humor	81	Undoing	111	Devalu_Other	141	Projection
22	Affiliation	52	React_Format	82	Devalu_Other	112	Projection	142	Splitting_Other
23	Self_Assertion	53	Intellectualizat	83	Undoing	113	Proj_Identific	143	Devalu_Other
24	Autis_Fantasy	54	Devalu_Other	84	Help_Rej_Com	114	Splitting_Self	144	Acting_Out
25	Affiliation	55	React_Format	85	Devalu_Other	115	Projection	145	Splitting_Other
26	Intellectualizat	56	Devaluat_Self	86	Rationalization	116	Passive_Aggr	146	Self_Assertion
27	Dissociation	57	Intellectualizat	87	Idealizat_Self	117	Suppression	147	Devaluat_Self
28	Isolat_Affect	58	Self_Observat	88	Passive_Aggr	118	Acting_Out	148	Autis_Fantasy
29	Devaluat_Self	59	Rationalization	89	Passive_Aggr	119	Humor	149	Help_Rej_Com
30	Dissociation	60	Intellectualizat	90	Self_Assertion	120	Rationalization	150	Suppression

**Label**	**Defense mechanism**				**Scoring**				

**Individual defense scores**									
D30	Suppression				[(Sum of items 49, 117, 128, 131, and 150) − 5]*100/234				
D29	Sublimation				[(Sum of items 14, 36, 63, 97, and 100) − 5]*100/234				
D28	Self-observation				[(Sum of items 9, 32, 58, 77, and 91) − 5]*100/234				
D27	Self-assertion				[(Sum of items 23, 90, 105, 109, and 146) − 5]*100/234				
D26	Humor				[(Sum of items 18, 37, 40, 51, and 119) − 5]*100/234				
D25	Anticipation				[(Sum of items 43, 46, 62, 65, and 78) − 5]*100/234				
D24	Altruism				[(Sum of items 11, 15, 79, 104, and 132) − 5]*100/234				
D23	Affiliation				[(Sum of items 22, 25, 44, 66, and 93) − 5]*100/234				
D22	Isolation of affects				[(Sum of items 28, 31, 39, 107, and 140) − 5]*100/234				
D21	Intellectualization				[(Sum of items 4, 26, 53, 57, and 60) − 5]*100/234				
D20	Undoing				[(Sum of items 48, 67, 70, 81, and 83) − 5]*100/234				
D19	Repression				[(Sum of items 13, 47, 50, 108, and 136) − 5]*100/234				
D18	Dissociation				[(Sum of items 8, 27, 30, 41, and 73) − 5]*100/234				
D17	React formation				[(Sum of items 52, 55, 74, 96, and 99) − 5]*100/234				
D16	Displacement				[(Sum of items 1, 64, 69, 122, and 125) − 5]*100/234				
D15	Devaluation other’s image				[(Sum of items 54, 82, 85, 111, and 143) − 5]*100/234				
D14	Devaluation self-image				[(Sum of items 12, 29, 34, 56, and 147) − 5]*100/234				
D13	Idealization other’s image				[(Sum of items 16, 17, 95, 138, and 139) − 5]*100/234				
D12	Idealization self-image				[(Sum of items 38, 71, 87, 133, and 135) − 5]*100/234				
D11	Omnipotence				[(Sum of items 7, 10, 68, 126, and 129) − 5]*100/234				
D10	Denial				[(Sum of items 20, 33, 121, 124, and 137) − 5]*100/234				
D9	Rationalization Sum of items 19, 42, 59, 86, and 120) − 5]*100/234				
D8	Projection				[(Sum of items 112, 115, 123, 134, and 141) − 5]*100/234				
D7	Autistic fantasy				[(Sum of items 2, 24, 106, 110, and 148) − 5]*100/234				
D6	Projective identification				[(Sum of items 72, 75, 101, 103, and 113) − 5]*100/234				
D5	Splitting of self-image				[(Sum of items 3, 6, 98, 142, and 145) − 5]*100/234				
D4	Splitting of object’s image				[(Sum of items 35, 61, 92, 94, and 114) − 5]*100/234				
D3	Passive aggression				[(Sum of items 45, 88, 89, 102, and 116) − 5]*100/234				
D2	Help-rejecting complaining				[(Sum of items 21, 84, 127, 130, and 149) − 5]*100/234				
D1	Acting out				[(Sum of items 5, 76, 80, 118, and 144) − 5]*100/234			

**Label**		**Defense level**				**Scoring**				

**Defense level scores**			
L7	High adaptive				Sum of D23, D24, D25, D26, D27, D28, D29, and D30				
L6	Obsessional				Sum of D20, D21, and D22				
L5	Neurotic				Sum of D16, D17, D18, and D19				
L5a	Hysterical				Sum of D18 and D19				
L5b	Other neurotic				Sum of D16 and D17				
L4	Minor image-distorting				Sum of D11, D12, D13, D14, and D15				
L3	Disavowal				Sum of D7, D8, D9, and D10				
L2	Major image-distorting				Sum of D4, D5, and D6				
L1	Action				Sum of D1, D2, and D3				

**Label**	**Defensive category**				**Scoring**				

**Defensive category scores**			
C3	Mature				Sum of D23, D24, D25, D26, D27, D28, D29, and D30				
C2	Neurotic				Sum of D16, D17, D18, D19, D20, D21, and D22				
C1	Immature				Sum of D1, D2, D3, D4, D5, D6, D7, D8, D9, D10, D11, D12, D13, D14, and D15				
C1a	Depressive				Sum of D1, D2, D3, D4, D5, D6, D8, D14, and D15				
C1b	Other immature				Sum of D7, D9, D10, D11, D12, and D13			

**Label**					**Scoring**				

**Overall defensive functioning**										
ODF					(L1/100)*1 + (L2/100)*2 + (L3/100)*3 + (L4/100)*4 + (L5/100)*5 + (L6/100)*6 + (L7/100)*7				

*For further information about the scoring system please contact the corresponding author.*

Although the scoring software has not yet been uploaded in the DMRS-Q web-app in order to protect it from hackers, we will include it after the publication of the present article. This upgrade will allow the DMRS-Q web-app to automatically calculate qualitative and quantitative scores after each evaluation and immediately deliver the DMRS-Q report to the user.

## Results

### The Defense Mechanisms Rating Scales Q-Sort Report

**FIGURE 3:**
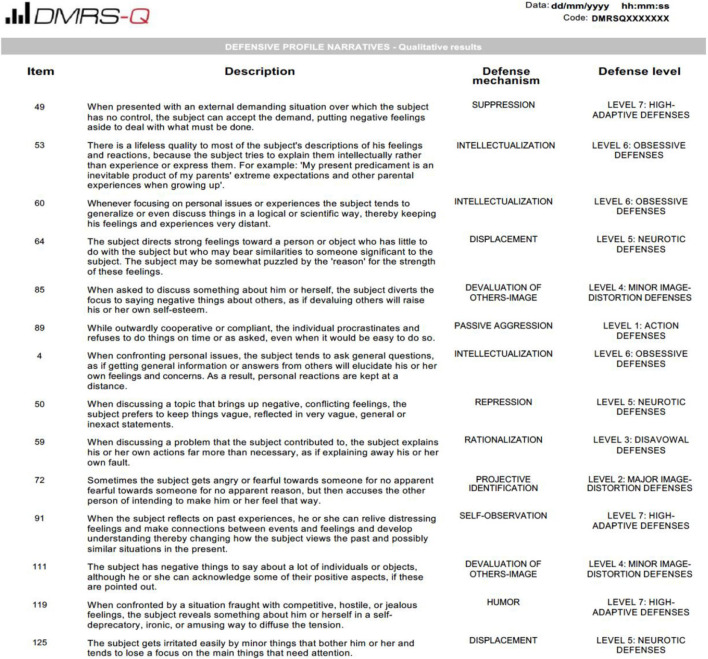
Defensive Profile Narrative (PDN) of a patient assessed with the DMRS.

Like the original DMRS, the DMRS-Q provides qualitative and quantitative scores reflecting the individual’s defensive functioning. Qualitative scores are displayed as the *Defensive Profile Narratives* (DPN), a case description of the most characteristic ways the subject handles internal conflict and external stressors. The DPN comprises all items sorted in ranks 6 and 7 (*N* = 14) and coded as highly descriptive of the subject’s defensive profile. The DMRS-Q software automatically lists these items and indicates the defense level and individual defense mechanism associated with each item. Figure 3 shows an example of a DPN displayed in the DMRS-Q report.

In addition to DPN, the DMRS-Q report provides the following quantitative scores: a summary Overall Defensive Functioning (ODF), ranging from 1 to 7; proportional scores for seven defense levels (see Table 1 for review); and proportional scores for 30 individual defense mechanisms (see Tables 2–8 for review). Future updates in the web-app software will also add scores for defensive categories and subcategories. Quantitative scores are displayed in both numerical and graphical forms in the DMRS-Q report, which can be downloaded from the user dashboard at any time.

### Clinical Vignette and Defense Mechanisms Rating Scales Q-Sort Rating

One example of how to use the DMRS-Q in clinical setting is offered by the following vignette. A brief description of patient-therapist interactions during the session is used for the DMRS-Q rating with no additional information about patient’s demographics, diagnosis, length of treatment, nor therapist’s approach, experience, etc. A summary of qualitative and quantitative evaluation of patient’s defense mechanisms analyzed with the DMRS-Q is displayed in Table 10. The 14 items coded as the best descriptive of the patient’s defensive functioning in the session were included in the qualitative defensive profile (DPN), while all item scores contributed to the quantitative scores displayed in the graphics.

**TABLE 10:** Qualitative and quantitative DMRS-Q evaluation of the described in the clinical vignette.

**Qualitative scores – Defensive profile narratives**
When confronted with topics that might be personally meaningful, the subject denies they are important and refuses to talk about them further. The subject complains spontaneously about how others don’t really care, or have made his or her problems worse, even when there is clear evidence that others have tried to help. At times when expressing an opinion or wish might be helpful, the subject fails to express himself adequately, instead finding indirect, even annoying ways to show his or her opposition to the influence of others, for example, being silent. The subject recites a litany of issues and problems but does not appear to be engaged in solving them, but rather prefers to complain. When others comment or inquire about the subject’s own feelings, actions, or intentions, the subject is very elusive or frankly denies the material, but the subject subsequently talks about similar feelings, actions, intentions, etc. in others. Whenever talking about potentially distressing events or experiences, the subject strongly claims not to have any feelings about the topic, although this seems highly unlikely. When telling an emotionally meaningful story, the subject states that he or she does not have specific feelings that one would expect, although the subject recognizes that he or she should feel something. In talking about a meaningful, emotionally charged experience, the subject talks in a detached way, as if he or she is not in touch with the feelings that should surround it. The subject avoids feelings of guilt or shame by justifying his actions or by referring to external reasons that impelled him to act. At times the subject’s feelings merge with those of another person and the subject assumes the other’s feelings and needs are exactly the same as the subject’s own. He or she then tends to ‘put words in the other’s mouth.’ Whenever confronted about his or her own feelings or intentions, the subject avoids acknowledging them by giving a plausible explanation that covers up the real subjective reasons. When angry toward someone significant, the subject takes anger out on himself instead of expressing it directly. The subject expresses hatred toward someone or something and refuses to acknowledge anything that does not confirm the hatred. When experiencing or confronted with a problem, the subject shames, humiliates, or blames someone else for the problem, ignoring his or her own role.
**Quantitative scores – Graphics**
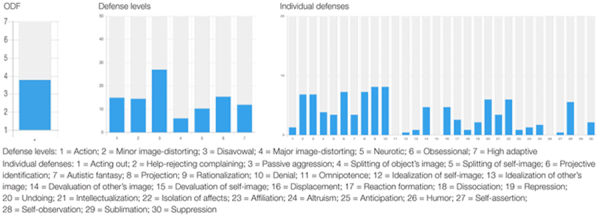

*The session started with the patient telling his negative experience with his lawyer and his attempt to solve a financial issue. While reporting on how the therapy had been helping him in enhancing his engagement in professional problems, the patient described himself with very devaluing terms. Even when the therapist tried to support him, saying that he was not aware of that difficulty, the patient made sarcastic comments toward the therapist and switched to another topic: the relationship with his girlfriend. The patient complained a lot about how frustrating this relationship was and justified his anger as the result of feeling too much pressure and low empathy at the same time. He made lots of devaluing comments about his girlfriend, although he could still see some positive aspects of her. Moreover, he reported on a series of passive aggressive behaviors toward a number of people (e.g., delay in return phone calls, calling up his ex-girlfriend, feeling bored in the session, feeling the therapist detached from him). Most of the session was characterized by the patient complaining about several aspects of his life, including the therapy, in which he had experienced ambivalence, detachment and frustration. When the therapist tried to interpret these feelings as defensive responses to the experience of a temporary unavailability of significant people, the patient denied the interpretation and perceived the therapist as manipulative. Despite therapist’s interpretations of his opposition, silence and emotional distancing as reactions to feeling frustrated by not getting what he wants when he wants, the patient rejected them and became even more oppositional. Toward the end of the session, after many therapist’s attempts of interpreting patient’s maladaptive pattern, the patient could finally reflect upon it and became more collaborative. However, his reflections were influenced by generalization, detachment and ambivalence. The patient described himself as stuck in silence, his inability to talk about his feelings, to see things in a different way. At this point the patient was able to let the therapist help him and get involved in a shared exploration of his fears, needs and desires. He reflected on his difficulty in listening to his girlfriend’s trouble but somehow justified it as a need of physical connection. However, when the therapist made further interpretations of the patient’s fantasy of emotional fusion, the patient seemed to reactivate the projective pattern, which was promptly interrupted by the therapist. This allowed the patient to keep reflecting in an ambiguous manner instead of complaining and activating all sorts of immature defense mechanisms.*

Table 10 displays PND and graphics of patient’s defensive functioning, including ODF, defense levels, and individual defenses scores. Defensive maturity fell in the range of severe depression or personality disorders (ODF < 4; Presniak et al., 2010; Perry and Bond, 2012; Di Giuseppe et al., 2019), with about 70% of immature defenses in use during the session, in particular those belonging to disavowal defense level. Looking at the use of individual defense mechanisms, the legend shows that patient’s predominant defenses were help-rejecting complaining, passive aggression, projecting identification, projection, rationalization, and denial. This defensive constellation indicates a depressive, resistant and passive aggressive patient inclined to withdraw inside himself and view his problems as externally caused, instead of dealing with his internal conflicts and external stressful situations.

## Discussion

The utility of studying defenses with the DMRS approach is that it reveals the psychological function behind the use of defense mechanisms, the unconscious motives for protecting oneself from intolerable emotional experiences. It could be the need of withdrawing anger, the threat of self-esteem failures, the shame of guilt experienced in confronting with unacceptable thoughts and many others. Any of these functions suggests what internal conflicts the individual is experiencing and how adaptive is his or her defensive functioning. In the present article we described the theoretical and methodological background of the DMRS-Q, illustrated its computerized and free-of-charge online use, provided directions for coding and described the interpretation of results.

While the assessment of defense mechanisms has been a controversial issue debated among scholars for more than a century, in recent years research, including that with the DMRS (Perry, 1990) convinced the American Psychiatric Association to include in the DSM-IV a provisional axis for the assessment of the hierarchy of defense mechanisms (American Psychiatric Association, 1994). However, the excellence of this highly valid and reliable method is unfortunately accompanied by its time-consuming training and coding costs, which led to the elimination of the defense axis in the DSM-5 because of lack of empirical findings supporting the theory (Vaillant, 1992).

With the development of the Q-sort version of the DMRS we provided a computerized and easy-to-use clinician-report measure for the assessment of the whole hierarchy of defense mechanisms observable in the routine practice of both dynamic and non-dynamic practitioners, as other have found (Starrs and Perry, 2018).

Apart from the well-established theory behind their development, the advantages of using this DMRS-based measure are numerous. First, the ODF score informs on how adaptive the individual’s defensive reaction is to internal conflicts and external stressful situations. This score can also be used as an outcome measure due to its strong correlation with other indexes of well-being. Second, the tripartite defensive category proportional scores tell to what extent the patient uses mature, middle-range and immature defenses. These scores are often used for a summary picture of the individual’s defensive functioning. Third, the seven defense level proportional scores reflect the prevalent defenses that have common functions at each level, and how much this contributes to ODF. Fourth, the 30 individual defense proportional scores provide a picture of the patient’s characteristic defense mechanisms, which reflects the most specific detailed level of defense assessment. These scores can capture differences between similar diagnostic categories, such as personality disorders (Maffei et al., 1995; Lingiardi and Giovanardi, 2017; Di Giuseppe et al., 2019, 2020d; Kramer, 2019), and reflect moment-to-moment micro-changes during the psychotherapy process (Hilsenroth and Pitman, 2019; Leibovich et al., 2020; Prout et al., 2021). Fifth, in addition to other DMRS measures (Perry, 1990; Di Giuseppe et al., 2020a), the DMRS-Q provides the patient’s defensive profile, a qualitative description of the most characteristic defensive patterns that contribute to determine the individual’s DPN (see the “Defensive Profile Narratives” in Table 10). Therapists can benefit from the use of all the above DMRS-Q scoring levels, in particular the individual defenses. These can guide therapeutic interventions to address desired changes in the patient’s defensive profile, thereby fostering therapeutic alliance and alleviating symptoms. Sixth, another remarkable quality of the DMRS-Q is its excellent support for teaching defense mechanisms. The use of simple examples of defensive responses provided by the DMRS-Q items, similar to the examples in the original DMRS Manual (Perry, 1990), can help the students’ understanding of definitions and functions of defense mechanisms. Moreover, the five items describing each defense mechanism can help in understanding differences in various occurrences of the same defense, especially the ones used uncommonly. Seventh, the main unique strengths of the DMRS-Q system are the short training required for its reliable use, the lack of necessity for transcriptions for coding defenses, and the free unlimited access to the DMRS-Q software from any electronic device connected to the internet. The estimated time for a DMRS-Q coding is approximately 15 min for expert trained raters who habitually code more than three sessions per week. This allows clinicians to code patients’ defense mechanisms after each session or a group of sessions and monitoring changes in defensive functioning during the therapeutic process (Wampold and Imel, 2015; Tanzilli et al., 2017, 2018, 2020).

The DMRS-Q has also some limitations that need consideration. First, the DMRS-Q is based on the Q-sort methodology, which requests the use of a *a priori* determined forced distribution that might limit the rater’s decision-making in the rank-ordering process. Second, the need for sufficient information on the patient’s defensive functioning is essential to ensure that the rater’s clinical inference for scoring all items into the forced distribution has an adequate evidentiary basis. Finally, the evaluation of defensive functioning is made on the overall defensive profile including all defensive phenomena observed. This methodology does not allow for the detection of specific defense mechanisms in use in particular moments, which is instead possible by applying the original DMRS to transcripts of clinical interviews or therapy sessions.

According to preliminary validation studies, the DMRS-Q seems a valid and reliable tool for the assessment of defense mechanisms in clinical settings, where the requirements for the use of the original DMRS are often unavailable (Di Giuseppe et al., 2014; Békés et al., 2021). A recent study (Békés et al., 2021) demonstrated that graduate students who received 6-h training reached excellent inter-rated reliability on the ODF (ICC = 0.90), good to excellent on defensive categories (ICC ranging from 0.83 to 0.92), and acceptable to excellent on the seven defense levels (ICC ranging from 0.74 to 0.92), with the only exception of major image-distorting defense level (ICC = 0.42) which is usually the less reliable scale due to the low base-rate of these defense mechanisms. On the other hand, non-trained students also showed excellent ICC on the ODF (ICC = 0.88) and acceptable to excellent on most DMRS-Q scales (ICC ranging from 0.60 to 0.91), except for the obsessional defense level (Békés et al., 2021). Good criterion validity was found in both clinical (Di Giuseppe et al., 2014) and community samples (Di Giuseppe et al., 2020a). Moreover, comparisons with mentalization and attachment showed great convergent and discriminant validity (Tanzilli et al., 2021). These results demonstrated that the DMRS-Q has very promising psychometric properties that must be confirmed by future studies on larger and more stratified samples.

## Conclusion

The systematic assessment of defense mechanisms in clinical settings is very important for monitoring the therapeutic process and aiding clinicians in choosing how to intervene in response to defenses used in the session (Fonagy et al., 2008; Gabbard, 2014; Conversano, 2021). The use of valid and reliable measures based on the gold-standard theory is essential for ensuring that what we observe is properly operationalized. The DMRS-Q is an easy-to-use, low-cost, computerized tool with promising psychometric properties can help clinicians in monitoring changes in defense mechanisms during the treatment, as suggested by others (Bhatia et al., 2017; Barber and Solomonov, 2019). The automatic scoring procedure provides a comprehensive report of qualitative and quantitative information on patient’s defensive functioning that can be used for clinical, research, and teaching purposes. The ease of use of the DMRS-Q makes this measure a potential candidate for fostering the observer-rated assessment of defense mechanisms in routine clinical practice and in process-outcome research.

## Data Availability Statement

The raw data supporting the conclusions of this article will be made available by the authors, without undue reservation.

## Author Contributions

The authors contributed in equal part to this work and approved it for publication. Both authors contributed to the article and approved the submitted version.

## Conflict of Interest

The authors declare that the research was conducted in the absence of any commercial or financial relationships that could be construed as a potential conflict of interest.

## Publisher’s Note

All claims expressed in this article are solely those of the authors and do not necessarily represent those of their affiliated organizations, or those of the publisher, the editors and the reviewers. Any product that may be evaluated in this article, or claim that may be made by its manufacturer, is not guaranteed or endorsed by the publisher.

## Footnotes

1
https://webapp.dmrs-q.com/login
